# A modular atomic force microscopy approach reveals a large range of hydrophobic adhesion forces among bacterial members of the leaf microbiota

**DOI:** 10.1038/s41396-019-0404-1

**Published:** 2019-03-20

**Authors:** Maximilian Mittelviefhaus, Daniel B. Müller, Tomaso Zambelli, Julia A. Vorholt

**Affiliations:** 1 Institute of Microbiology, ETH Zurich, Zurich, Switzerland; 2 Institute for Biomedical Engineering, ETH Zurich, Zurich, Switzerland

**Keywords:** Environmental microbiology, Applied microbiology

## Abstract

Bacterial adhesion is the initial step in surface colonization and community formation. At the single-cell level, atomic force microscopy (AFM) techniques have enabled the quantification of adhesive forces between bacteria and substrata. However, conventional techniques depend on the irreversible immobilization of cells onto cantilevers, thus hampering throughput. Here, we developed a modular AFM method to reversibly immobilize functionalized beads as surface mimic and to probe adhesion of individual bacteria. We performed single-cell force spectroscopies with phylogenetically diverse leaf isolates of various size and morphology. Adhesion measurement of 28 bacterial strains revealed large differences in hydrophobic interactions of about three orders of magnitude. The highest adhesion forces of up to 50 nN were recorded for members of the Gammaproteobacteria. The hydrophobicity of the different isolates correlated positively with the retention of bacteria observed *in planta* and might provide a basis for successful leaf colonization and potentially disease outbreaks of pathogens.

Bacteria colonize their habitats in complex multicellular communities. Adhesion of bacteria to a substratum constitutes the initial step for colonization and development of complex networks of microorganisms on surfaces [1, 2]. Bacterial adhesion can be divided into two phases. Initial attachment is driven by physicochemical forces between bacteria and substratum such as van der Waals, electrostatic, and hydrophobic interactions, while in the second phase molecular-specific interactions become more important and strengthen the adhesion [3].

Through development of single-cell force spectroscopy techniques, atomic force microscopy has emerged as a valuable tool to study bacterial adhesion [4]. While offering single-cell resolution of adhesion forces, these approaches are limited by the time consuming generation of cell probes, resulting in the use of one cantilever per measured cell. Bacteria are irreversibly immobilized onto a cantilever and subsequently brought into contact with a surface of choice before retracting the cantilever to record the forces from the cantilevers deflection.

Fluidic force microscopy (FluidFM) enables the reversible immobilization of micro-objects, including cells, at the aperture of a microchanneled cantilever by aspiration [5, 6]. By direct aspiration of single bacterial cells, the adhesion of *Escherichia coli*, *Streptococcus pyogenes*, and *Caulobacter crescentus* has been successfully probed [7, 8]. To accommodate different bacterial sizes and morphologies, the apertures of the microfluidic probes can be adapted by focused ion beam milling [7]. However, if a large panel of bacteria are screened to measure adhesion forces, individual adaptation to cell morphology and size would require a large effort and a straightforward method to screen different microorganisms with minimal sample preparation is desirable.

Here, we demonstrate a modular workflow allowing us to quantify the adhesion forces of phylogenetically diverse bacteria to a surface that mimics their natural substrate. We reversibly immobilize functionalized silica beads on the FluidFM cantilever [9] to probe the hydrophobic interaction of a representative collection of bacterial isolates from *Arabidopsis thaliana* leaves [10]. As the latter are coated by epicuticular waxes that are predominantly composed of alkanes with carbon chain lengths of 29–31 atoms [11], we chose C30-functionalized beads to mimic the hydrophobic leaf surface.

During bacterial adhesion measurement, the setup is operated in aqueous buffer and a negative pressure is applied to reversibly immobilize a bead on the tipless aperture of the cantilever. Target bacteria are immobilized onto a glass surface by polydopamine coating to prevent displacement of cells during the measurement (Fig. 1a) [12, 13]. The bead is brought into contact with an isolated, viable cell – a process that is monitored under simultaneous optical inspection using an inverted microscope. After reaching a defined force, here 10 nanoNewton (nN), the contact is maintained for 5 s before retracting the cantilever and recording its deflection, caused by the occurring adhesive forces between bead and bacterium (Fig. 1b). The bead is then used again to measure the adhesive force to another bacterial cell; alternatively, the bead is exchanged by expelling it through a short overpressure pulse and a new bead is picked up readily with the same cantilever (see Supplementary Material and Methods).

**Fig. 1:**
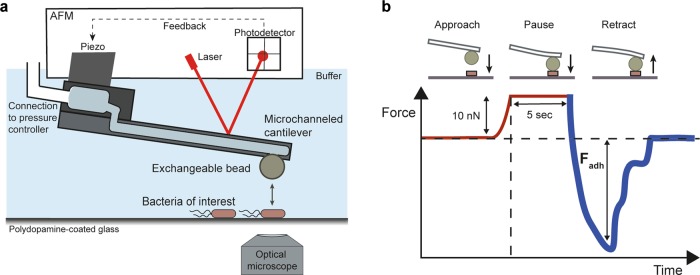
FluidFM-based single-cell force spectroscopy using functionalized beads. **a** Schematic representation of the experimental setup. A functionalized silica bead is reversibly immobilized by application of a negative pressure through the microchanneled cantilever. The bead is used to probe for interactions with bacteria that are immobilized on polydopamine-coated glass. A laser beam reflected on the back of the cantilever serves to monitor the forces acting on the cantilever. The feedback control between the photodetector and the piezo element allows precise application of defined forces as well as detection of adhesion forces. **b** Workflow of adhesion measurements. The cantilever is deflected due to the forces acting between bacterium and bead over the time of the experiment. Adhesion forces (F_adh_) were derived from the maximally measured force during the retraction phase. After each measurement the immobilized bead is used again for another cell or exchanged

For our analysis, we chose 26 bacterial strains from a strain-collection previously isolated from the leaves of *A. thaliana* [10], the leaf commensal *Sphingomonas melonis* Fr1 [14] and *E. coli*. The leaf strains span all four of the main bacterial phyla that constitute the microbiota of *A. thaliana*, and feature diverse cell morphologies (Figs. 2a, S1). Highlighting the universal applicability of our method, we were able to quantify and compare the hydrophobic adhesion for all strains, ranging from the short rod-shaped *Plantibacter* L1 (0.7 × 1.2 µm) to the chain-forming *Bacillus* L13 (1.4 × 3.7 μm). To ensure clean surfaces, we regularly exchanged beads during measurement, using at least three different beads per bacterial strain tested. We did not observe any systematic error between different beads or a significant reduction in the measured forces when probing up to 14 individual bacterial cells with the same bead (Figs. S2, S3).

**Fig. 2:**
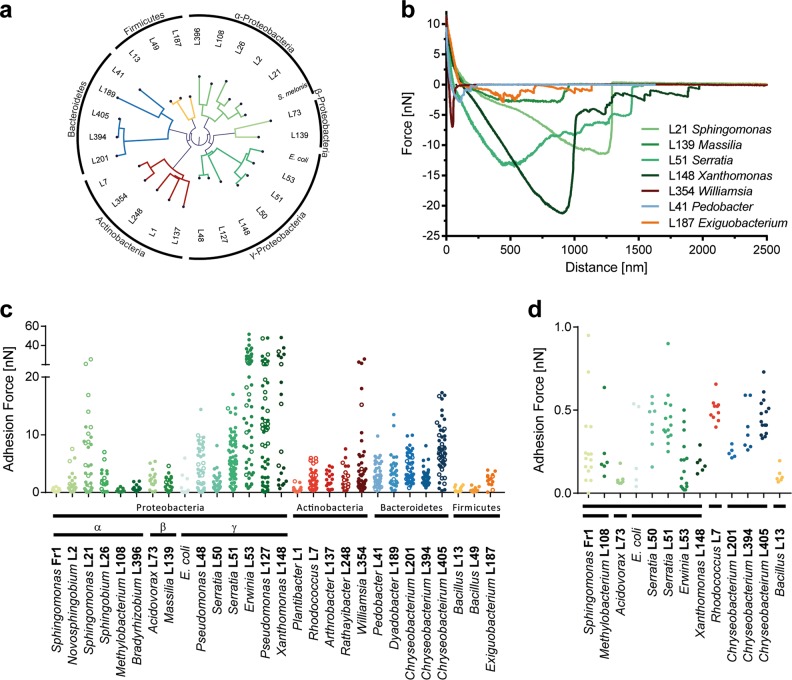
Adhesion forces of bacterial leaf isolates. **a** Phylogenetic tree of bacteria isolated from *Arabidopsis thaliana* leaf-isolated bacteria used in this study. **b** Representative retraction force curves of the interaction between leaf isolates and hydrophobic beads (C30). Adhesion forces of individual bacterial cells towards C30-functionalized (**c**) or plain (**d**) silica beads. Each symbol depicts the maximal adhesion force recorded when retracting the bead from a single cell. Open symbols represent force spectroscopies during which the bacterium was detached from the underlying polydopamine coating. **c** Different strains (28 in total) were probed for their adhesion to hydrophobic beads. At least 10 single-cell force spectroscopies were performed for each strain. **d** Representative strains (13 in total) of all four bacterial phyla were tested for their adhesion to plain silica beads. For each strain at least five cells were measured

The collected retraction profiles showed differences in the magnitude of adhesion forces as well as distinct force patterns, some of which were indicative of cellular appendages in both Gram-positive and Gram-negative cells (i.e., the force jumps in *Exiguobacterium* L187 or *Xanthomonas* L148; Figs. 2b, S4, S5). These characteristic patterns follow the maximal adhesion peak and are defined by a phase of increasing force or a force-plateau, prior to a sharp reduction of the recorded force. Such force jumps are caused by the successive stretching and rupture of adhesive bonds. These might be formed for example by pili as shown for *Pseudomonas aeruginosa* or *Lactobacillus rhamnosus*, where these force patterns were absent from retraction profiles of isogenic mutants lacking type IV pili or SpaCBA pili, respectively [15, 16]. Due to their protruding nature, cellular appendages may serve to bridge larger distances in attachment to a substratum [17]. For example, flagella have been shown to increase bacterial adhesion to hydrophobic substrates [18] and can increase bacterial attachment to surfaces by enabling cells to reach into crevices that are inaccessible to the bacterial cell body [19].

Covering all 26 leaf isolates and reference bacteria, we measured more than 700 individual cells, revealing a broad spectrum of adhesion forces to the hydrophobic beads (Fig. 2c). Adhesion forces were derived from retraction profiles as the maximally recorded force (Fig. 1b). The highest forces of up to 50 nN were measured for members of the Gammaproteobacteria, especially from the genera *Pseudomonas*, *Erwinia*, and *Xanthomonas*, which harbor some of the most important bacterial phytopathogens [20]. Notably, pathogenic species of *Erwinia* and *Xanthomonas* have been proposed to depend on efficient adhesion for formation of mature biofilms and plant colonization [21, 22]. The adhesion forces measured here are in good agreement with previously reported values for single-cell force spectroscopies. A study using single-cell probes reported adhesion forces to glass surfaces below 1 nN for *E. coli*, 1–2 nN for *Pseudomonas fluoresce*ns, and 10–15 nN for *Staphylococcus epidermis* [23], while another study reported forces up to 50 nN for the interaction between individual *Bacillus mycoides* spores and hydrophobic surfaces [24]. In some of our measurements, the interaction between bacterium and bead exceeded the immobilization strength of the bacterium on the polydopamine-coated glass, effectively limiting the measurable force (Fig. 2c open symbols). The resulting detachment was evident from optical monitoring of the cell during force spectroscopy as well as the shape of retraction profiles, which showed sharp drops of the recorded forces back to baseline (Fig S6). Since such events prevented measurement of the true hydrophobic adhesion forces, we excluded these values from further analysis. Demonstrating the hydrophobic nature of the recorded interactions, an assessment of C18-functionalized beads yielded comparable, albeit generally lower adhesion forces of exemplary isolates than C30-functionalized beads (Fig S7). In marked contrast, members of all four phyla displayed only weak adhesion of 1 nN or lower when tested against unfunctionalized silica beads, with differences among the strains likely due to nonspecific electrostatic or van der Waals forces (Fig. 2d) [3].

The throughput of our method compared to conventional AFM-based single-cell force spectroscopy allowed us to measure larger cell populations and to observe single-cell heterogeneity, a feature often masked in population-level analyses or the use of only a limited number of cell probes [25]. Bacterial cell surface hydrophobicity is inherently heterogeneous and differences among sister cells may arise due to dynamic changes during the cell cycle or different regulatory responses to contact with the underlying substratum [26–28]. Considering that attachment of a few cells, followed by clonal expansion, might be sufficient to colonize a surface efficiently, this heterogeneity might prove beneficial to bacterial populations [29]. Subpopulations exhibiting lower hydrophobic adhesion forces may be better equipped to adhere to different substrates or relocate to more favorable sites for example.

To investigate whether the hydrophobic interaction forces correlate with the attachment behavior *in planta* we performed washing assays with 30-day old *A. thaliana* plants. We submerged the aboveground part of individual plants in mixed bacterial suspensions of varying complexity for 10 min before washing the plants in sterile water [30]. Based on amplicon sequencing of 16S rRNA genes, we determined the relative abundances of bacterial strains retained on the plant after washing compared to the initial suspension (Supplementary Material and Methods, Supplementary Table 1; Fig. 3). Comparing the retention with the median of measured single-cell adhesion forces indicated that higher adhesion forces towards C30-beads correlated positively with increased initial retention on plants when using a mixture of 215 leaf-associated bacterial isolates [10] as inoculum (Spearman correlation *r*_s_ = 0.5528, *P* = 0.0034). We also repeated the experiment with an inoculum composed of only 25 bacterial strains and again found a positive correlation between our adhesion measurements and initial retention on leaf surfaces (Spearman correlation *r*_s _= 0.5168, *P* = 0.0116). In addition, the correlation between hydrophobic forces and retention *in planta* was confirmed by a plating assay with three closely related Proteobacteria strains L50 *Serratia*, L51 *Serratia*, and L53 *Erwinia* (Fig S8).

**Fig. 3:**
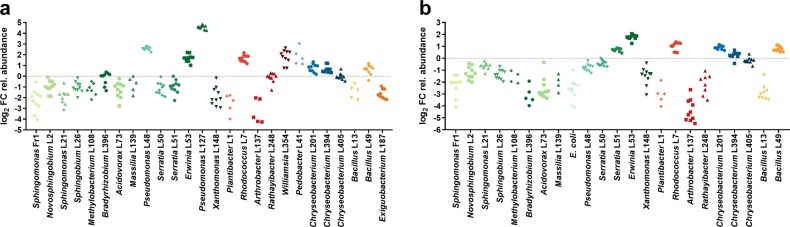
Retention of bacteria on plant surfaces. Initial retention of each individual strain on plant surfaces after inoculation and washing compared to the original suspension (depicted here as log_2_-transformed fold change in relative species abundance). Each symbol depicts one of 10 replicate plants. Suspensions of either 215 leaf isolates (**a**) or 24 leaf isolates as well as *E. coli* (**b**) were used as inocula for plant retention assays. Shown are the 26 (**a**) or 23 (**b**) strains that were present in all inoculum samples and that were probed for hydrophobic adhesion in single-cell force spectroscopy experiments

In conclusion, we developed and applied a versatile method to quantify adhesion of diverse bacterial cells. Due to the modularity of the approach, it is readily adaptable to quantify interactions of interest. Our results corroborate the role of hydrophobicity in initial attachment of bacteria to their natural host, here leaves; however, the distinct features employed by bacterial species to achieve this as well as the influence of surface patterning remain to be elucidated. Likewise, it will be of interest to decipher the cause and consequence of the observed heterogeneity.

## Supplementary information

Supplemental Material
