# Supplementary material for: A modular atomic force microscopy approach reveals a large range of hydrophobic adhesion forces among bacterial members of the leaf microbiota
Source: ISME J. 2019 Mar 20;13(7):1878–82. doi: 10.1038/s41396-019-0404-1 (PMC6591122; doi:10.1038/s41396-019-0404-1)
Supplement: Supplementary file 1 — Supplemental Material [file 41396_2019_404_MOESM1_ESM.pdf]

Supplementary Information

A modular atomic force microscopy approach reveals a large range of hydrophobic adhesion forces among bacterial members of the leaf microbiota

Maximilian Mittelviefhaus, Daniel B. Müller, Tomaso Zambelli, Julia A. Vorholt

Content

Supplementary Material and Methods

Supplementary Figures S1-S8

Supplementary Table 1

Supplementary References

Supplementary Material and Methods

Bacterial strains

Bacterial leaf isolates (1), *Sphingomonas melonis* Fr1 (2), and *E. coli* DH5α were grown on R2A agar (Sigma-Aldrich, St. Louis, MO, USA) plates at room temperature. Genomes of all leaf isolates are available in BioProject accession PRJNA297956. For force spectroscopy experiments, bacterial colonies were scraped off the surface of R2A agar plates with a inoculation loop and resuspended in PBS (9.6 g/l NaCl, 0.24 g/l KCl, 1.73 g/l Na_2HPO_4, 0.28 g/l KH_2PO_4, in distilled water, pH 7.4) before immobilization in glass-bottom microscopy dishes (WillCo Wells, Amsterdam, Netherlands). To facilitate targeting of individual bacteria as well as ensuring measurement of live cells, a viability staining (Live/Dead® BacLight™, Molecular Probes, Eugene, OR, USA) was included during sample preparation according to the manufacturer's instructions. Analogously, for washing assays on plants, either 215 or 25 bacterial strains were scraped off the agar plates and resuspended in PBS, pooled in roughly equal ratios, and adjusted to a final OD_{600} of 0.5. For single-strain washing assays L50 *Serratia*, L51 *Serratia*, or L53 *Erwinia* were resuspended in PBS individually and also adjusted to an OD_{600} of 0.5.

Growth conditions of *Arabidopsis thaliana* plants

Seeds of *A. thaliana* Col-0 were surface sterilized as described previously (3). For washing assays with mixed bacterial inocula, plants were cultivated in full-gas microboxes (Combiness, Nevele, Belgium) with sterile lumox® film 25 (Sarstedt, Nümbrecht, Germany)) on the surface of the solidified MS medium (Duchefa Biochemie, Haarlem, Netherlands) (4), following the protocol of Innerebner *et al.* (2). Eight plants per microbox were grown in growth chambers (CU-41L4, Percival Scientific, Perry, IA, USA) under long-day conditions (16 hours light/day) for 1 week and then switched to short-day conditions (9 hours light/day). For single-strain washing assays, plants were grown under the same conditions but in 24-well plates, according to Vogel *et al.* (5).

Bacterial immobilization for force spectroscopy

To immobilize bacteria on the microscopy dish during force spectroscopy experiments, they were immobilized on polydopamine-coated glass (6). Glass-bottom microscopy dishes (WillCo Wells) were coated with polydopamine (Sigma Aldrich) as previously described (7). Briefly, glass dishes were cleaned by sonication in 2-propanol (Sigma Aldrich) and ultra-pure water before drying under N_2-stream and subsequent plasma treatment for 2 min (Plasma Cleaner PD-32G, Harrick Plasma, Ithaca, NY, USA). Polydopamine (4 g/l in 10 mM TRIS HCl, pH 8.5) was applied to the cleaned glass surface for 1 hour before washing extensively with PBS. Bacteria resuspended in PBS were allowed to interact with the coated surface for 60 min. Unbound bacteria were washed off by rinsing with PBS and the adherent bacteria were covered with 4 mL PBS to ensure immersion of the FluidFM setup in liquid.

FluidFM setup

The FluidFM setup was composed of a FlexAFM scanhead, C3000 controller (both Nanosurf AG, Liestal, Switzerland), and a digital pressure controller (Cytosurge AG, Glattbrugg, Switzerland). The scanhead was mounted on top of an AxioObserver Z1 inverted microscope (Carl Zeiss, Jena, Germany) to allow for optical control throughout the experiments. Beads and bacteria were targeted and imaged using LD Plan-Neofluar 40x/0.6 or EC Plan-Neofluar 100×/1.30 oil immersion objectives and an AxioCamMR (Carl Zeiss).

Cantilever preparation and calibration

Tipless hollow cantilevers (Cytosurge AG) with a nominal spring constant of 0.2 N/m and a 2 µm aperture at their distal end, were used for bacterial adhesion measurements. Prior to use, cantilevers were plasma cleaned for 30 s (Plasma Cleaner PD-32G, Harrick Plasma) and coated

with SL2 Sigmacote (Sigma Aldrich) to prevent fouling; probes were kept in a desiccator containing 1 ml of Sigmacote siliconizing reagent overnight and subsequently dried at 100 °C for 60 min. The spring constant of cantilevers was determined based on its resonance frequency in air using Nanosurf C3000 software (Nanosurf AG) (8). The microchannel inside the cantilever was filled with filtered ultra-pure water by application of pressure using a digital pressure controller (Cytosurge AG). The cantilever sensitivity was recalibrated after every exchange of beads.

Force spectroscopy procedure

Single cell force spectroscopies were performed at room temperature in PBS (pH 7.4). 5 µm large C30-functionalized (Reprospher 100 C30-DE 5 µm, Dr. Maisch GmbH, Ammerbuch, Germany), C18-functionalized (Reprospher 100 C18-DE 5 µm, Dr. Maisch GmbH), or plain silica beads (sicastar plain, micromod Partikeltechnologie GmbH, Rostock, Germany) (all 100 Å pore size and 350 m^2/g specific surface area) were dispersed onto a confined region of the glass dish that did not feature any surface coating to prevent soiling of the beads. Cantilevers were brought into contact with selected silica beads with a force-setpoint of 20 nN. During contact, a negative pressure of 800 mbar was exerted to reversibly immobilize the bead at the cantilever aperture. Remaining in liquid, the cantilever with the immobilized bead was moved to a region of the glass dish harboring immobilized bacteria. Optical microscopy was used to target an isolated and viable cell (indicated by green fluorescence after Live/Dead-staining as described above). The selected bacterium was approached with the bead-probe at a speed of 1 µm/s until a force of 10 nN was recorded. To allow steady interaction, this force was kept constant for 5 s, before retracting the bead-probe at a piezo velocity of 1 µm/s while recording occurring forces. Adhesion forces were derived from force distance curves as the maximally measured forces along the profile, using SPIP software (Image Metrology A/S, Hørsholm, Denmark). During each force spectroscopy, optical microscopy was used to ensure that the targeted cell had not detached from the underlying glass

dish during measurement. In case of detachment, the measurement was excluded from further analysis and a new bead was used for the next measurements. Otherwise, multiple cells were measured with one immobilized bead; however, at least three separate beads were used for every bacterial strain tested. For each strain, at least two independently grown batches of bacteria were prepared and a minimum of 10 individual cells was measured.

Washing Assay

For washing assays with mixed bacterial inocula, axenic *A. thaliana* plants grown in microboxes were harvested at 30 days age and the roots were removed using a sterilized scalpel immediately before the experiment. Care was taken to select similarly sized individual plants. Per inoculum, 10 individual plants were placed in separate wells of 12-well plates and submerged in 2 mL of bacterial suspension. After 10 min the plants were taken from the suspension and washed in 40 mL of sterile water by vigorously agitating the plant using sterile tweezers for 15 s (9). This washing step was repeated with fresh water. After shaking off excess liquid, plants were transferred individually to Lysing Matrix E tubes (MP Biomedicals, Illkirch, France) and frozen at -80 °C. Plants treated analogously but submerged in PBS were included as axenic controls. To determine the relative abundances of the individual strains in the inocula used, 500 µL were transferred directly to Lysing Matrix E tubes (MP Biomedicals) and centrifuged for 5 min at 10 000 rpm. After removing the resulting supernatant the inoculum samples were also frozen at -80 °C.

Based on 16S rRNA genes, relative abundances of bacterial strains remaining on leaves after washing as well as in the original inocula, were determined as described previously (1). Briefly, samples were homogenized and DNA was extracted using the FastDNA SPIN Kit for soil (MP Biomedicals) according to the manufacturer's protocol. DNA concentrations were quantified with the Quantifluor® dsDNA system (Promega, Madison, WI, USA) before amplifying 16S rRNA genes by targeting the variable regions V5-V7 (primers 799F and 1193R, see Supplementary

Table 1). After removal of remaining primers and nucleotides, a second PCR elongated the amplicons by introduction of barcodes specific for each sample (Forward primers B5-F3 or B5-F4 and one of 96 reverse primers B5-1 to B5-96, see Supplementary Table 1). After barcoding, bacterial 16S rRNA gene amplicons were recovered by extraction from agarose gel (QIAquick Gel Extraction Kit, Qiagen, Hilden, Germany) and cleaning with the AMPure XP Kit (Beckman Coulter, Indianapolis, IN, USA). Sequencing was performed on a Miseq platform using a Miseq reagent kit v3 and 2 x 300 bp paired-end protocol (all Illumina, San Diego, CA, USA). Using scripts from the QIIME pipeline, reads were joined, demultiplexed, and quality filtered (Phred ≥20) (10). Based on a reference database with the 16S rRNA genes of all used strains, the reads were then assigned to the respective strains using a similarity threshold of 100 %. Relative abundances were calculated as the number of reads for a specific strain over the total number of reads in the corresponding sample. Strains that were not present in every technical replicate of the respective inoculum were excluded from further analysis. The ratio of relative abundance of a given strain in the sample compared to that in the inoculum was used as measure for the initial retention on the plant surface. Spearman's rank correlation coefficients between the median adhesion forces towards C30-beads and the initial retention on plants were calculated in GraphPad Prism (GraphPad Software, La Jolla, CA, USA).

Analogously, we performed washing assays with single strains. Harvest, inoculation, and washing were performed as described above. The fresh weight of washed plants was determined and they were transferred to tubes containing 1.3 mL sodium phosphate buffer (100 mM, pH 7), supplemented with 0.2% Silwet L-77 surfactant (Leu+Gygax AG, Birmenstorf, Switzerland). Bacteria retained on the leaf surface were washed off by 15 min of intense shaking (25 Hz, TissueLyser II, Qiagen, Hilden, Germany), followed by sonication in a water bath for 5 min as described by Innerebner *et al.* (2). Serial dilutions of washed off bacterial cells were spotted on R2A agar and colony-forming units were enumerated after growth at room temperature. The experiment was performed with three independent bacterial cultures.

Supplementary Figures

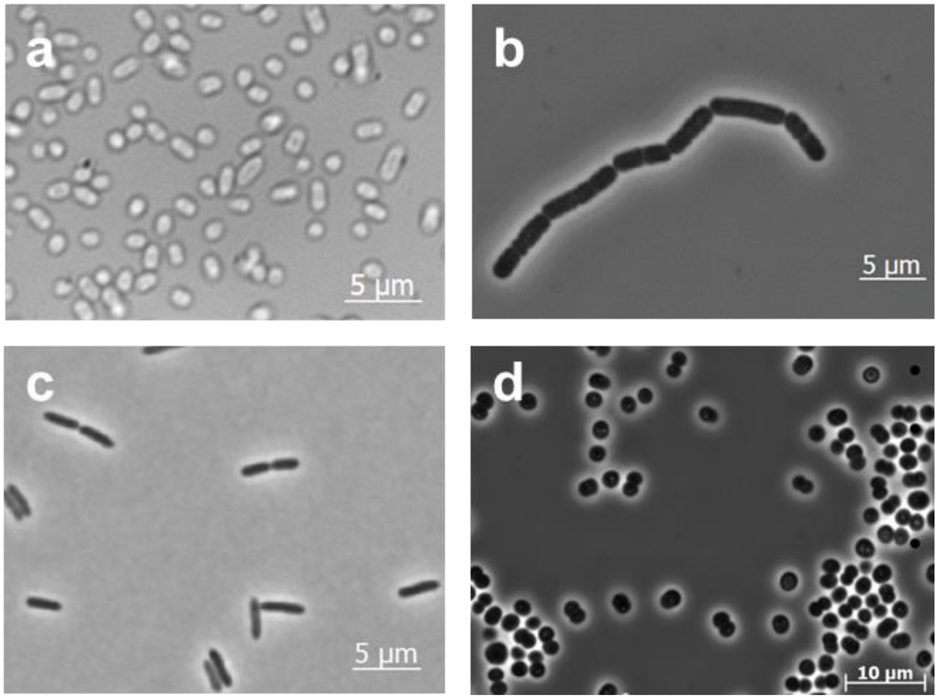

Supplementary Figure S1. Exemplary micrographs of tested bacterial species. Microscopy images of (a) Leaf53 *Erwinia*, (b) Leaf13 *Bacillus*, (c) Leaf148 *Xanthomonas*, and (d) L137 *Arthrobacter*.

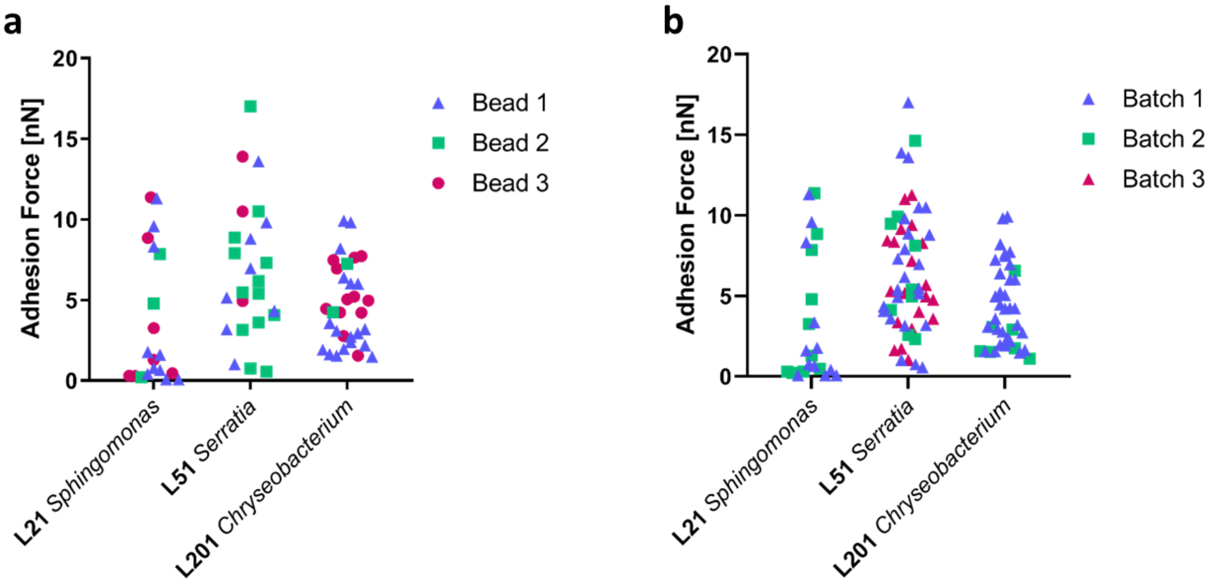

Supplementary Figure S2. Adhesion measurements using different beads and batches of bacteria. (a) Three distinct C30-functionalized beads were used to probe adhesion of each bacterial strain. Symbols depict the interaction between an individual cell and different beads, represented by different shape and color of the symbols. No significant effect (One-way ANOVA) between the different beads was observed. (b) Two or three independent cultures of bacteria were used for adhesion measurements (depicted by color). Symbols depict the interaction between an individual cell and C30-functionalized beads. No significant effects (One-way ANOVA for L51; t-test for others) between the replicate biological cultures were observed.

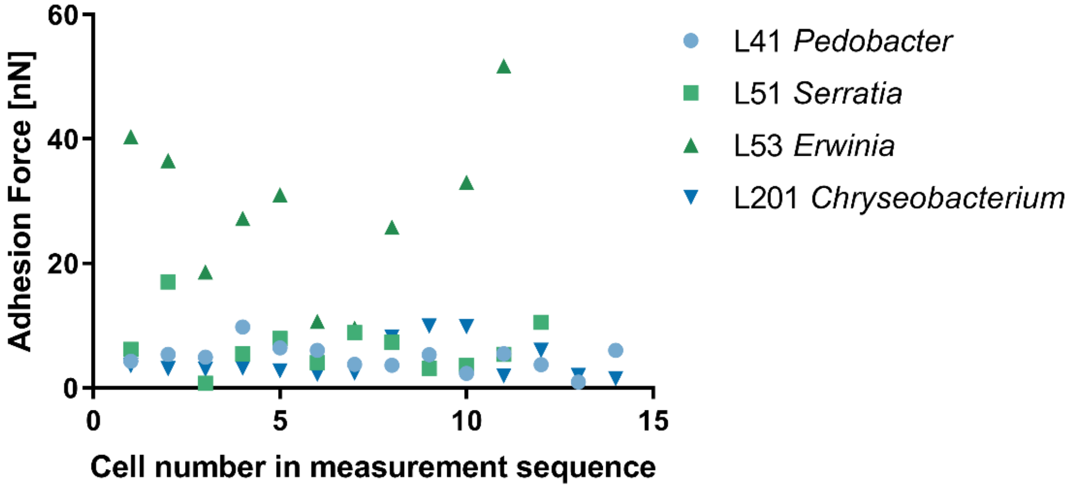

Supplementary Figure S3. Repeated adhesion measurements using the same bead. One bead was applied for sequential measurement of multiple cells. The sequence of measurements followed the indicated cell number. Up to 14 contacts between bead and different bacteria did not result in a significant reduction or increase (Spearman correlation) of the measured adhesion forces.

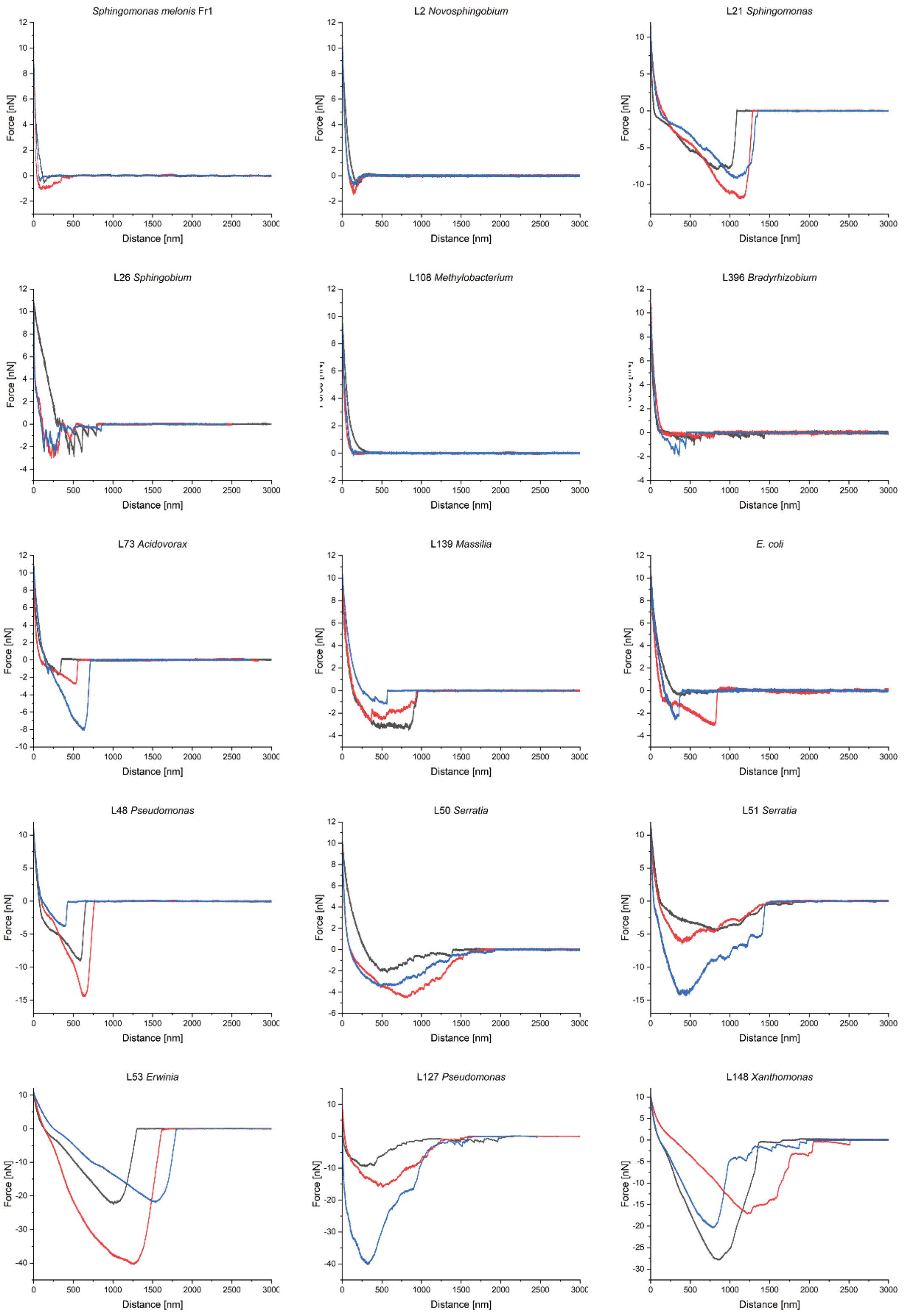

Supplementary Figure S4. Retraction profiles of the interaction between Proteobacteria leaf isolates and C30 beads. Three representative force profiles of distinct bacterial cells are given for each strain.

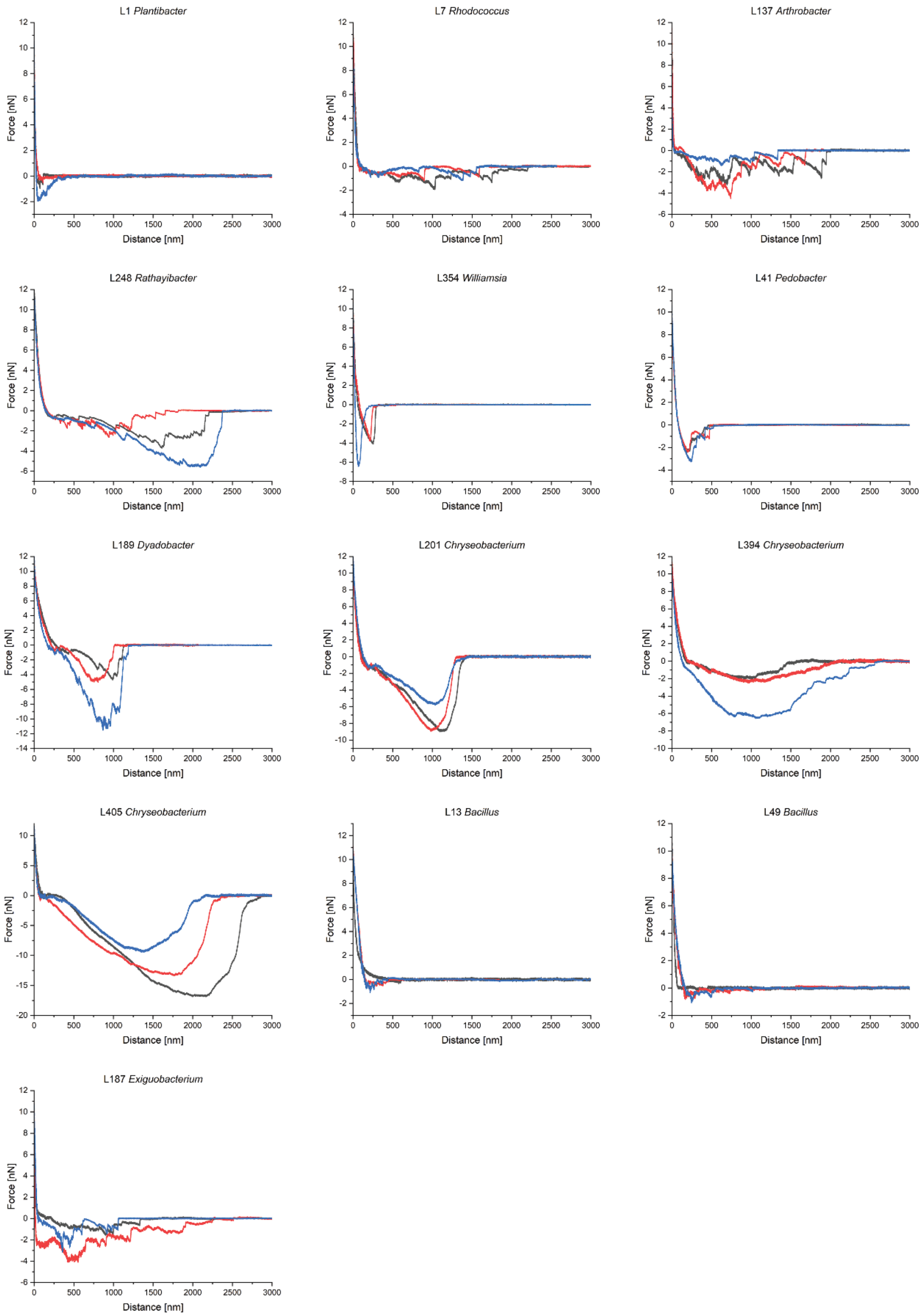

Supplementary Figure S5. Retraction profiles of the interaction between Actinobacteria, Bacteroidetes and Firmicutes leaf isolates and C30 beads. Three representative force profiles of distinct bacterial cells are given for each strain.

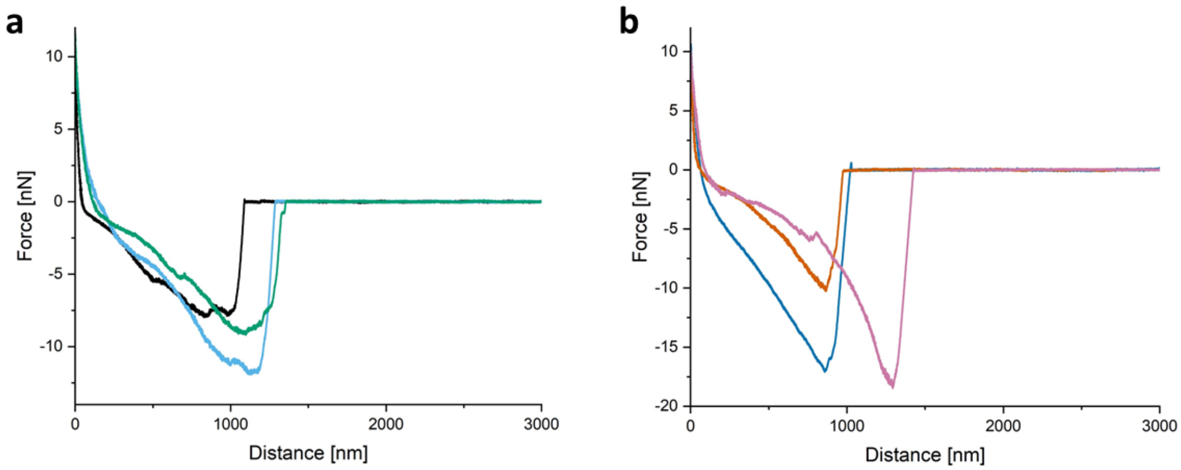

Supplementary Figure S6. Exemplary retraction profiles of the interaction between C30 beads and L21 *Sphingomonas*. During force spectroscopy, bacterial cells (a) stayed attached to the polydopamine-coated glass surface or (b) were detached from it. Shown are retraction profiles for three individual cells for each case.

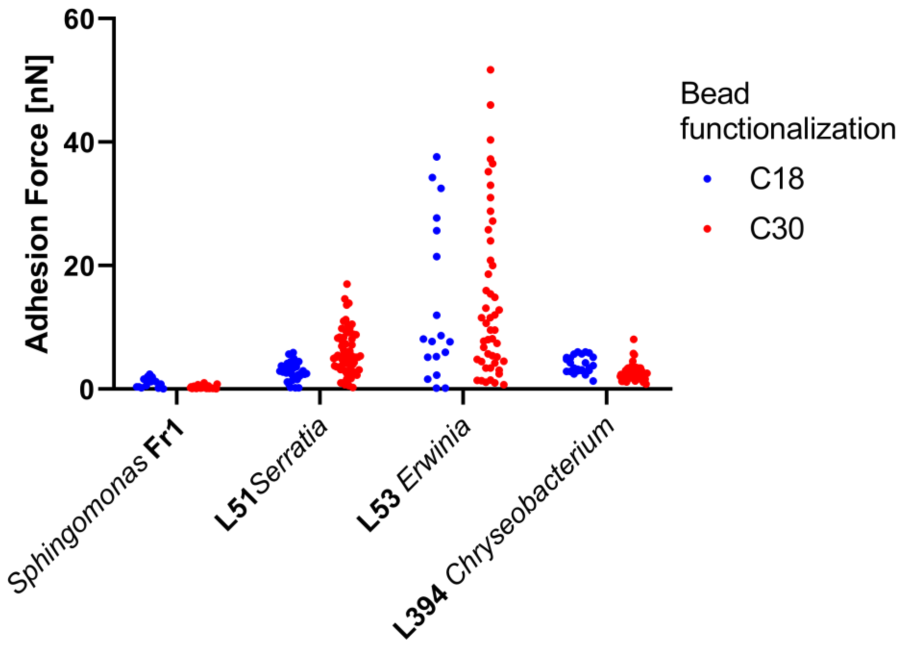

Supplementary Figure S7. Comparison of adhesion forces towards C18- and C30-functionalized silica beads. Each symbol represents the maximal adhesion force recorded during single-cell force spectroscopy.

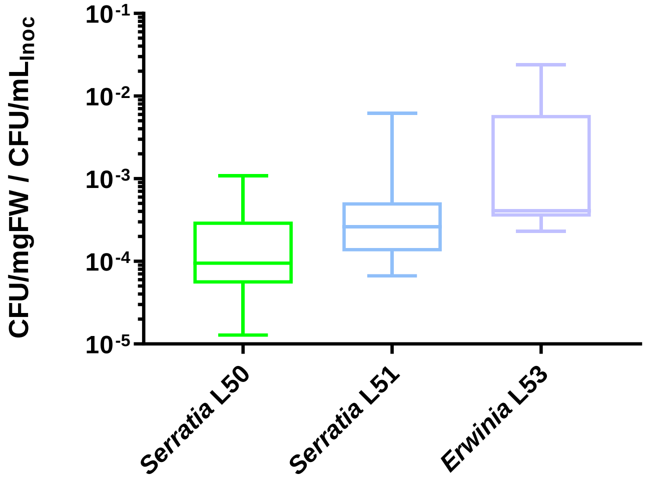

Supplementary Figure S8. Retention of individually inoculated bacterial strains during washing assays. Bacterial colony-forming units per milligram of plant fresh weight (CFU/mgFW) retained on *A. thaliana* leaves as compared to the used inocula (CFU/mL_{Inoc}). Lines depict the median, boxes extend between 25th and 75th percentile, and whiskers depict minimal and maximal values. The experiment was repeated independently three times.

Supplementary Table

Supplementary table 1. Primers used for PCR amplification of 16S rRNA gene amplicons. Bases introducing sample-specific barcodes are underlined.

Primer	Sequence (5'->3')	Ref.
799F	AACMGGATTAGATACCCKG	(1)
1193R	ACGTCATCCCCACCTTCC	(1)
B5-F3	AATGATACGGCGACCACCGAGATCTACACGTCTGGACTGCGACTGGC GAACMGGATTAGATACCCKG	
B5-F4	AATGATACGGCGACCACCGAGATCTACACCAGTGGACTGCGACTGG CGAACMGGATTAGATACCCKG	
B5-1 to B5-96	CAAGCAGAAGACGGCATACGAGATXXXXXXXXXXXXCAGCCATTTAGT GTCACGTCATCCCCACCTTCC	(1)

Supplementary References

1. Bai Y, Müller DB, Srinivas G, Garrido-Oter R, Potthoff E, Rott M, et al. Functional overlap of the *Arabidopsis* leaf and root microbiota. *Nature* 2015; 528(7582): 364–9.
2. Innerebner G, Knief C, Vorholt JA. Protection of *Arabidopsis thaliana* against leaf-pathogenic *Pseudomonas syringae* by *Sphingomonas* strains in a controlled model system. *Appl Environ Microbiol* 2011; 77(10): 3202–10.
3. Schlesier B, Bréton F, Mock HP. A hydroponic culture system for growing Arabidopsis thaliana plantlets under sterile conditions. *Plant Mol Biol Report* 2003; 21(4): 449–56.
4. Murashige T, Skoog. A revised medium for rapid growth and bioassay with tobacco tissue cultures. *Physiol Plant* 1962; 15: 473–97.
5. Vogel C, Innerebner G, Zingg J, Guder J, Vorholt JA. Forward genetic *in planta* screen for identification of plant-protective traits of *Sphingomonas* sp. strain Fr1 against *Pseudomonas syringae* DC3000. *Appl Environ Microbiol* 2012; 78(16): 5529–35.
6. Lee H, Dellatore SM, Miller WM, Messersmith PB. Mussel-inspired surface chemistry for multifunctional coatings. *Science* 2007; 318(5849): 426–30.
7. Potthoff E, Ossola D, Zambelli T, Vorholt JA. Bacterial adhesion force quantification by fluidic force microscopy. *Nanoscale* 2015; 7(9): 4070–9.
8. Sader JE, Chon JWM, Mulvaney P. Calibration of rectangular atomic force microscope cantilevers. *Rev Sci Instrum* 1999; 70(10): 3967–9.
9. Leben C, Whitmoyer RE. Adherence of bacteria to leaves. *Can J Microbiol* 1979; 25(8): 896–901.
10. Caporaso JG, Kuczynski J, Stombaugh J, Bittinger K, Bushman FD, Costello EK, et al. QIIME allows analysis of high-throughput community sequencing data. *Nat Methods* 2010; 7(5): 335–6.
